# Mapping real-time metabolic kinetics of expanded CAR T cells using hyperpolarized ^13^C-glucose and metabolomics

**DOI:** 10.1038/s41598-025-24712-2

**Published:** 2025-11-20

**Authors:** Thomas B. Wareham Mathiassen, Mikkel Rasmus Hansen, Magnus Karlsson, Sine Reker Hadrup, Maria Ormhøj, Pernille Rose Jensen

**Affiliations:** 1 Section for Magnetic Resonance, Department of Health Technology, Technical University of Denmark, 2800 Kgs. Lyngby, Denmark; 2 Section for Experimental and Translational Immunology (xTI), Department of Health Technology, Technical University of Denmark, 2800 Kgs. Lyngby, Denmark; 3 Section for Biotechnology, Department of Green Technology, University of Southern Denmark, Odense, Denmark

**Keywords:** CAR T cells, Metabolism, NMR spectroscopy, dDNP-NMR, Immunotherapy, Biochemistry, Immunology

## Abstract

Chimeric Antigen Receptor (CAR) T cell therapy has advanced cancer treatment, providing options for patients with refractory hematological malignancies. Understanding the metabolic changes in CAR T cells during their activation, proliferation, and therapeutic application is essential for improving efficacy. In this study, human CD19 CAR T cells were expanded from three healthy donors under a standard manufacturing protocol. Hyperpolarized ^13^C dDNP-NMR spectroscopy was used to measure glycolytic flux at key time points (days 1, 7, 14, and 21), and ^1^H NMR metabolomics was employed to monitor nutrient depletion in the culture medium. The results showed that CAR T cells underwent a metabolic transition from oxidative phosphorylation to aerobic glycolysis by day 7, followed by a return to oxidative phosphorylation by day 21. Glucose depletion was most pronounced during the first week, whereas amino acids were substantially consumed, with a decline in consumption observed after day 12. Real-time NMR spectroscopy revealed large metabolic flux changes that metabolomics did not capture. This study demonstrates the dynamic metabolic plasticity of CAR T cells and highlights the utility of hyperpolarized ^13^C-NMR for tracking glycolytic activity. Addressing metabolic bottlenecks, such as nutrient depletion during early expansion, may improve CAR T cell manufacturing and therapeutic outcomes.

**Supplementary Information:**

The online version contains supplementary material available at 10.1038/s41598-025-24712-2.

## Introduction

Immunotherapy, using T cells genetically modified to express a chimeric antigen receptor (CAR) has shown tremendous potential in treating hematological cancers^1,2^. Despite extensive effort, the initial success of CAR T cell therapy has not been recapitulated to the same extent in the context of solid tumors^3^. In solid tumors, barriers to effective CAR T cell therapy include limited trafficking to the tumor site, lack of *in vivo* persistence, and impaired efficacy due to the immunosuppressive factors and metabolic constraints imposed by a hostile tumor microenvironment (TME)^4^. These constraints, especially competition for nutrients like glucose, limit the function of effector T cells^3^. T cells, as major effectors of the adaptive immune system, play a critical role in combating pathogens and cancer. Upon recognizing specific antigens, T cells rapidly alter their metabolic state, switching from oxidative phosphorylation to aerobic glycolysis, a process known as the Warburg effect where cells predominantly convert glucose into lactate rather than fully oxidizing it via the tricarboxylic acid (TCA) cycle^5–7^. This shift facilitates rapid ATP synthesis and supports the high proliferation rate needed to amass the cell numbers required for an effective immune response. Specific activation through T cell receptor (TCR) and CD28 signaling regulates glycolysis in activated T cells, partly through the phosphoinositide 3-kinase (PI3K)-dependent expression of glucose transporter 1 (GLUT1)^7,8^. As the immune response progresses, activated T cells gradually reduce lactate production and shift back toward oxidative phosphorylation. Once the infection is resolved, many of the expanded T cells die off, leaving behind a subset of memory T cells in a metabolic maintenance state^9,10^. This metabolic plasticity is crucial for the effective function and long-term persistence of T cells, ensuring a robust and adaptable immune response.

CAR T cell therapy involves ex vivo genetic modification and expansion of patient T cells before re-infusion of an adequate number of CAR T cells into the patient^11^. The ex vivo expansion of the CAR T cells is thus a crucial part of the workflow^12^. Recent findings have shown that T cells with lower glycolytic rates and higher oxidative phosphorylation exhibit enhanced persistence, cytotoxicity, and overall functionality^13,14^. Despite renewed interest in the metabolic state of T cell products, most studies often rely on indirect metabolic assays, such as the Seahorse assay using the extracellular acidification factor (ECAR) and oxygen consumption rate (OCR), to monitor the metabolic state of T cells in relation to their glycolytic rates and oxidative phosphorylation^15,16^. Nuclear Magnetic Resonance (NMR) spectroscopy is a fast, unbiased, and versatile analytical method for measuring cell metabolism. Most commonly NMR metabolomics is used to determine the composition of metabolites in biological samples^17^ and for cell cultures the nutrient availability and exo metabolome can be measured directly on the growth medium giving specific information on major metabolites such as amino acids, glucose and lactate^18^. Sensitivity enhanced NMR via dissolution dynamic nuclear polarization (dDNP) introduces a physical labelling on a chosen substrate which allows real-time flux measurements through metabolic pathways, providing detailed insights into cellular metabolism^19^. The method involves polarizing isotopically labeled substrate (^13^C) in a supercooled polarizer (1.3 K) using a strong magnet and microwaves to transfer polarization to the nuclei^20^. The sample is then dissolved in buffer and delivered to cells in an NMR spectrometer. The method provides five orders of magnitude enhancement on the chosen substrate, compared to standard NMR techniques^20^. The vast signal enhancement enables real-time, non-invasive monitoring of substrate conversion for about two minutes. Pyruvate is the most common substrate for hyperpolarized NMR. It is converted to lactate by the enzyme lactate dehydrogenase (LDH) in the final step of glycolysis and is often used as an indicator of anaerobic respiratory activity. To date, hyperpolarized pyruvate has been used to measure the activity of LDH in activated T cells, which revealed a 3.6-fold increase in pyruvate to lactate conversion in 5-day activated T cells compared to non-activated T cells^21^. Metabolomic studies using mass spectrometry have shown that T cells undergo significant metabolic reprogramming upon activation to support proliferation and effector function. Findings show increased glucose uptake and glycolysis, glutamine-driven TCA cycle anaplerosis, and altered fatty acid and lipid metabolism. Additionally, changes in nucleotide synthesis and activation of mTOR-driven metabolic pathways are essential for T cell activation^22–24^.

Detailed understanding of T cell metabolism during expansion can provide insights into their functional capabilities and potential vulnerabilities, which is essential for developing the most robust protocols for T cell therapy.

In this study, we combined NMR metabolomics with dDNP-NMR to monitor the metabolic state of CAR T cells over a 21-day expansion protocol. This approach provided insights into the metabolic dynamics during CAR T cell expansion. Real-time glycolytic flux measurements with hyperpolarized [U-^13^C,^2^H]glucose revealed a surprisingly high metabolic plasticity in CAR T cells with a more than 30-fold difference between minimum at day 1 and maximum at day 7. Metabolomic analysis of the growth medium revealed glucose depletion within the first 10 days of the expansion protocol, accompanied by substantial amino acid consumption. Incorporating NMR metabolomics directly on cell culture media offers a rapid and cost-effective addition to current analytical techniques for characterizing CAR T cell expansion. Additionally, dDNP-NMR flux measurements provide a promising method for advancing metabolic assessments and quality control during CAR T cell manufacturing.

## Results

### Establishing hyperpolarized [U-^13^C,^2^H]glucose for non-invasive measurement of glycolytic flux

**Fig. 1:**
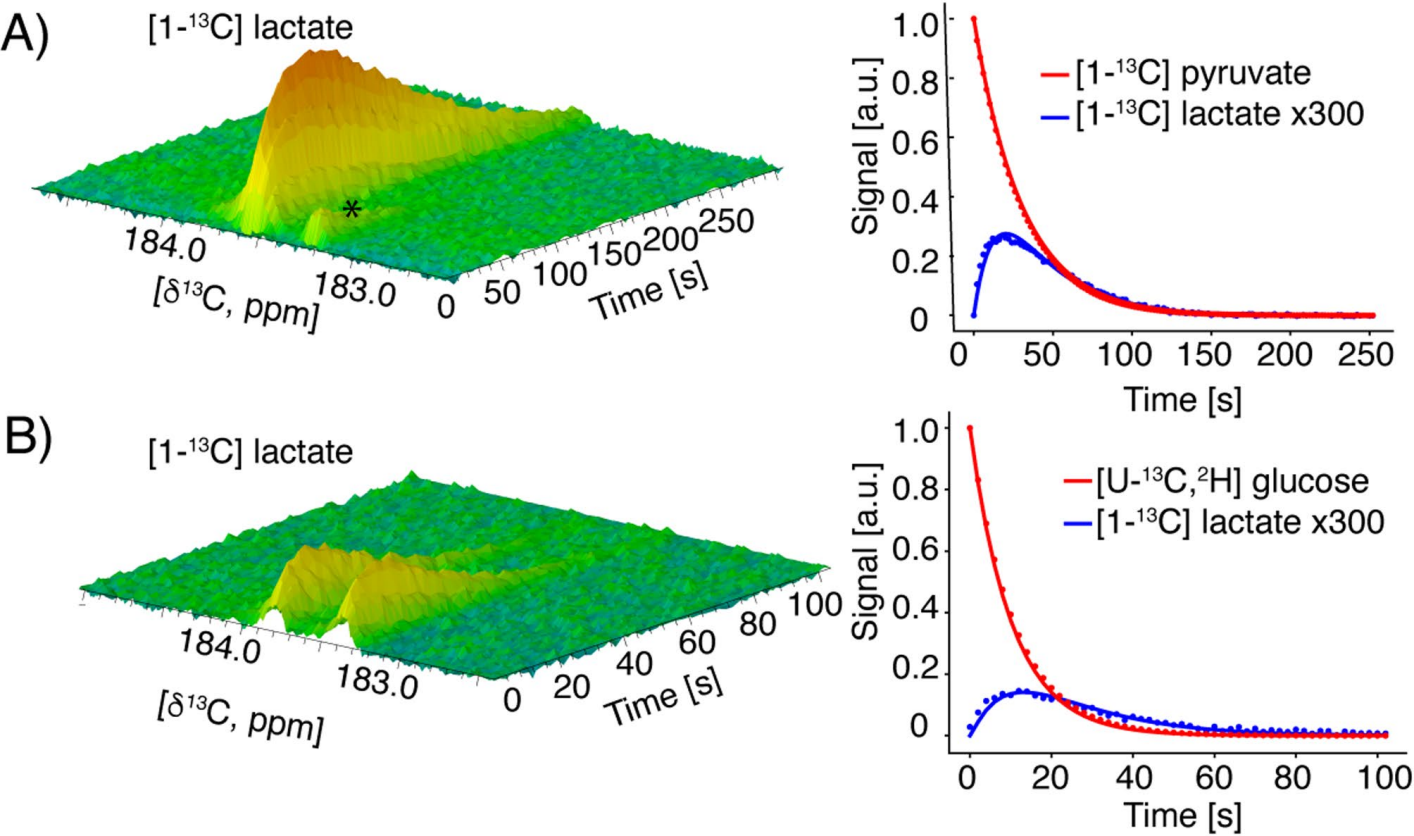
T Cell Metabolism: T cell metabolism measured with hyperpolarized substrates after five days of activation with CD3/CD28 Dynabeads^™^ and IL-2. (**A**) Pyruvate-derived lactate production from hyperpolarized [1-^13^ C]pyruvate, with a fitted rate constant of 10.7 × 10^-5^ s^-1^. (* impurity from [1-^13^ C]pyruvate, fit R^2^ = 0.999 and 0.991 for substrate and product, respectively), and (**B**) glucose-derived lactate production over time in seconds derived from hyperpolarized [U-^13^C,^2^H]glucose, with a rate constant of 9.4 × 10^-5^ s^-1^ [1-^13^ C]lactate from [U-^13^C,^2^H]glucose is split in a doublet due to ^1^*J* ^13^C-^13^C coupling from the uniform labelled substrate (^1^*J* ^13^C-^13^C = 55 Hz, fit R^2^ = 0.999 and 0.978 for substrate and product, respectively).

Human T cells were isolated from a healthy donor buffy coat sample using EasySep^™^ Human T cell Separation beads. The T cells were then activated using CD3/CD28 Dynabeads^™^ and IL2 and the cells were expanded in RPMI medium as floating cells. Every second day the cells were counted and diluted to 0.5 × 10^6^ cells/mL. At day 5 after activation 10 × 10^6^ cells were harvested by centrifugation and transferred to an NMR spectrometer. Hyperpolarized [1-^13^C]pyruvate was injected into the T cell suspension to monitor the metabolic flux. The hyperpolarized [1-^13^C]pyruvate was metabolized directly to [1-^13^C]lactate and the apparent rate constant was determined to 10.7 × 10^−5^ s^−1^ by fitting the data to a set of linear differential equations describing the signal evolution as function of metabolic conversion, signal relaxation (T1) and pulsing (Fig. 1A)^25^. To minimize the number of fitted parameters the contribution of flip angle was included in the T1 decay which resulted in apparent T1s of 30 and 16 s for [1-^13^C]pyruvate and [1-^13^C]lactate (flip angle 8°, TR = 2 s), respectively. The observed conversion of [1-^13^C]pyruvate was consistent with a previous study, which showed that hyperpolarized [1-^13^C]pyruvate effectively tracked metabolic changes in CD4 T lymphocytes activated for five days^21^. Next, we evaluated whether hyperpolarized [U-^13^C,^2^H]glucose could provide additional insights into T cell glycolysis. Hyperpolarized [U-^13^C,^2^H]glucose was injected into 10 × 10^6^ T cells activated for five days and conversion to [1-^13^C]lactate was observed (Fig. 1B). Due to the inherent^1^*J* ^13^C-^13^C coupling in the fully labelled glucose the lactate signal was split into a doublet compared to [1-^13^C]lactate produced from [1-^13^C]pyruvate. The corresponding apparent glycolytic rate constant was determined to 9.4 × 10^-5^ s^-1^ by fitting the data to a set of linear differential equations. Apparent T1 of 10 and 18 s were fitted for [U-^13^C,^2^H]glucose and [1-^13^C]lactate (flip angle 15°, TR = 2 s), respectively. The conversion from [1-^13^C]pyruvate to [1-^13^C]lactate is only one enzymatic step whereas the rate constant for [U-^13^C,^2^H]glucose to [1-^13^C]lactate covers all steps in the glycolysis. Therefore, the calculated rate constants are not directly comparable. The maximal height of the [1-^13^C]lactate peak was similar using the two metabolic probes, [1-^13^C]pyruvate and [U-^13^C,^2^H]glucose. Since [U-^13^C,^2^H]glucose provided similar signal to noise for the product, this substrate was chosen for further metabolic studies as it was desirable to measure flux from the full glycolysis and not only the last enzymatic step, as was the case with [1-^13^C]pyruvate.

### Metabolic characterization of CAR T cells during full expansion protocol

**Fig. 2:**
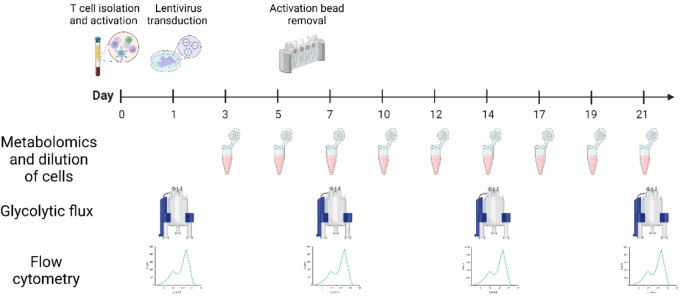
Schematic of CAR T cell expansion protocol and sampling strategy. Cultures were generated from three healthy donors. T cells were isolated from donor buffy coat samples and activated using CD3/CD28 Dynabeads^™^ and IL-2 at 1 × 10^6^ cells/mL. On day 1 the T cells were infected with Lentivirus containing a CD19 CAR. On day 3, supernatants were sampled for ^1^H NMR and the cell diluted to 0.5 × 10^6^ cells/mL. This procedure was repeated every second day. Beads were removed on day 6, and IL-2 supplementation followed medium change. Cells were collected on day 1, 7, 14, and 21 for flow cytometry and dDNP-NMR analysis.

A study protocol was designed to provide a metabolic investigation of CAR T cells through a 21-day expansion protocol following a standard CAR T cell activation protocol^26,27^ (Fig. 2). In brief, T cells were isolated from three different donor buffy coat samples and seeded at a concentration of 1 × 10^6^ cells/mL on day 0 while stimulating them with CD3/CD28 Dynabeads^™^ and IL-2. On day 1 T cells were transfected with lentivirus containing an anti-CD19 CAR at an MOI of 5. From this point onward the cells were diluted to 0.5 × 10^6^/mL every second day. On day 1, 7, 14, and 21, cells from each donor were collected for hyperpolarized NMR analysis and flow cytometry. Additionally, samples for ^1^H NMR metabolomics analysis were taken from the cell growth medium each time the cells were diluted.

**Fig. 3:**
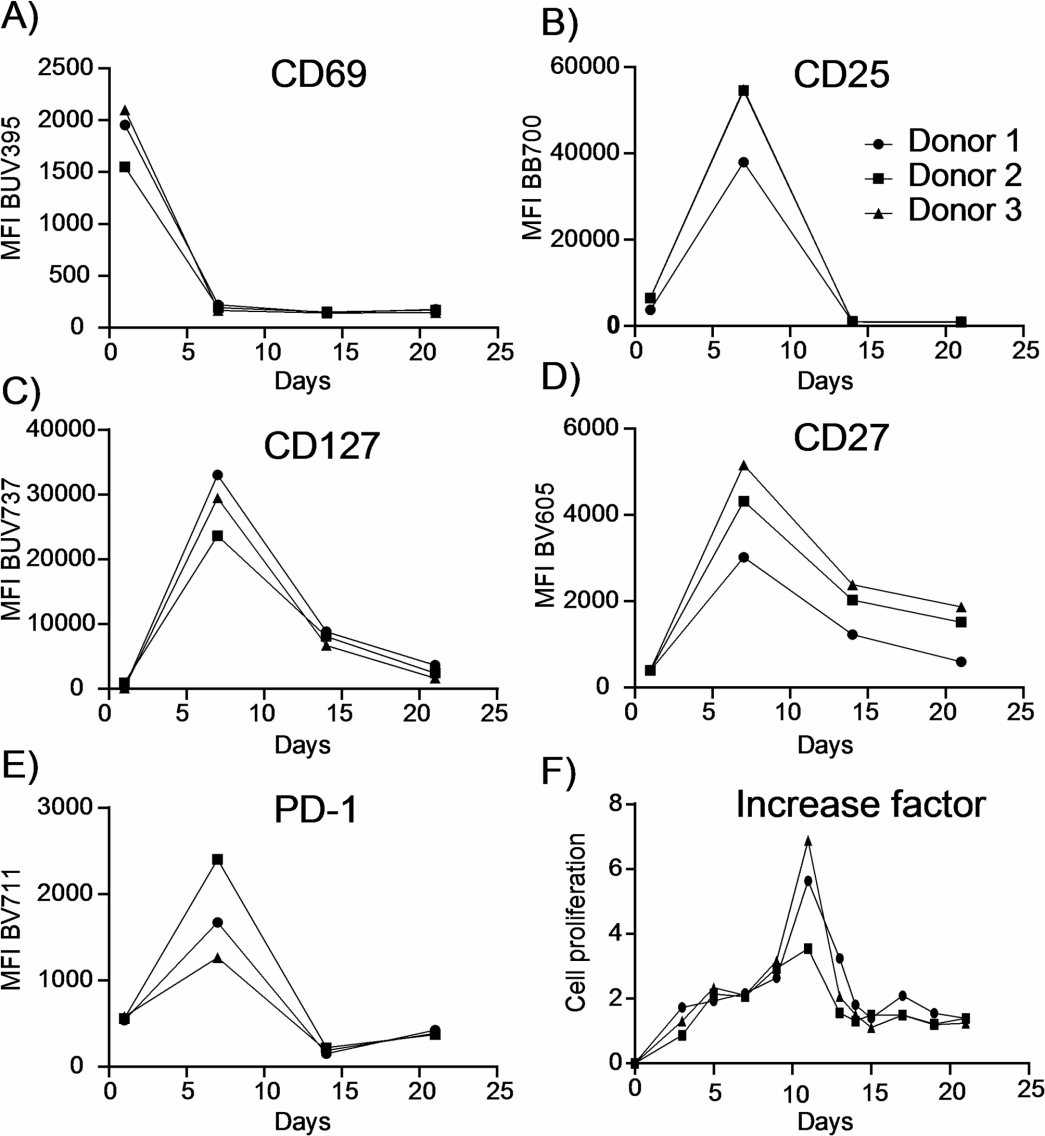
Expression of CAR T cell activation markers. Activation markers CD69, CD25, CD127, CD27, and PD-1 in CAR T cells were measured using flow cytometry over a 21-day expansion period. The median fluorescence intensity (MFI) reflected the relative expression levels of each marker, based on the fluorescence emitted by the antibody-fluorochrome conjugates used to stain the cells. Cell proliferation is represented as the population increase factor, calculated as: Population Increase Factor = (new cell count – previous cell count) / previous cell count.​ *N* = 1 for three individual donors.

Expression of CAR T cell activation markers was determined with flow cytometry on days 1, 7, 14, and 21, as shown in Fig. 3. The early activation marker CD69, an immediate-early lectin receptor, was shown to initially be expressed after 24 h. Later activation markers were detected on day 7, including CD25 the high-affinity IL-2 receptor alpha subunit linked to proliferation, CD127 the IL-7 receptor associated with T cell survival, CD27 a co-stimulatory molecule promoting expansion, and PD-1, a checkpoint molecule regulating exhaustion. These markers have earlier been shown to be upregulated within some days after activation^28^. Cell proliferation peaked on day 11 after activation, following the expected trajectory observed in other CAR T cell studies, such as Kawalekar et al.^28^. Additionally, we quantified CAR expression by flow cytometry and observed a gradual reduction in CAR-positive cells over time, suggesting that recombinant protein expression may impose a metabolic burden. CD4/CD8 subset frequencies confirmed that our measurements were performed in a clinically relevant CAR T cell mixture (Fig. S1). These results confirm expected activation and proliferation thereby providing a solid foundation for interpreting the metabolic data.

### Real-time metabolic dynamics in CAR T cells during expansion

**Fig. 4:**
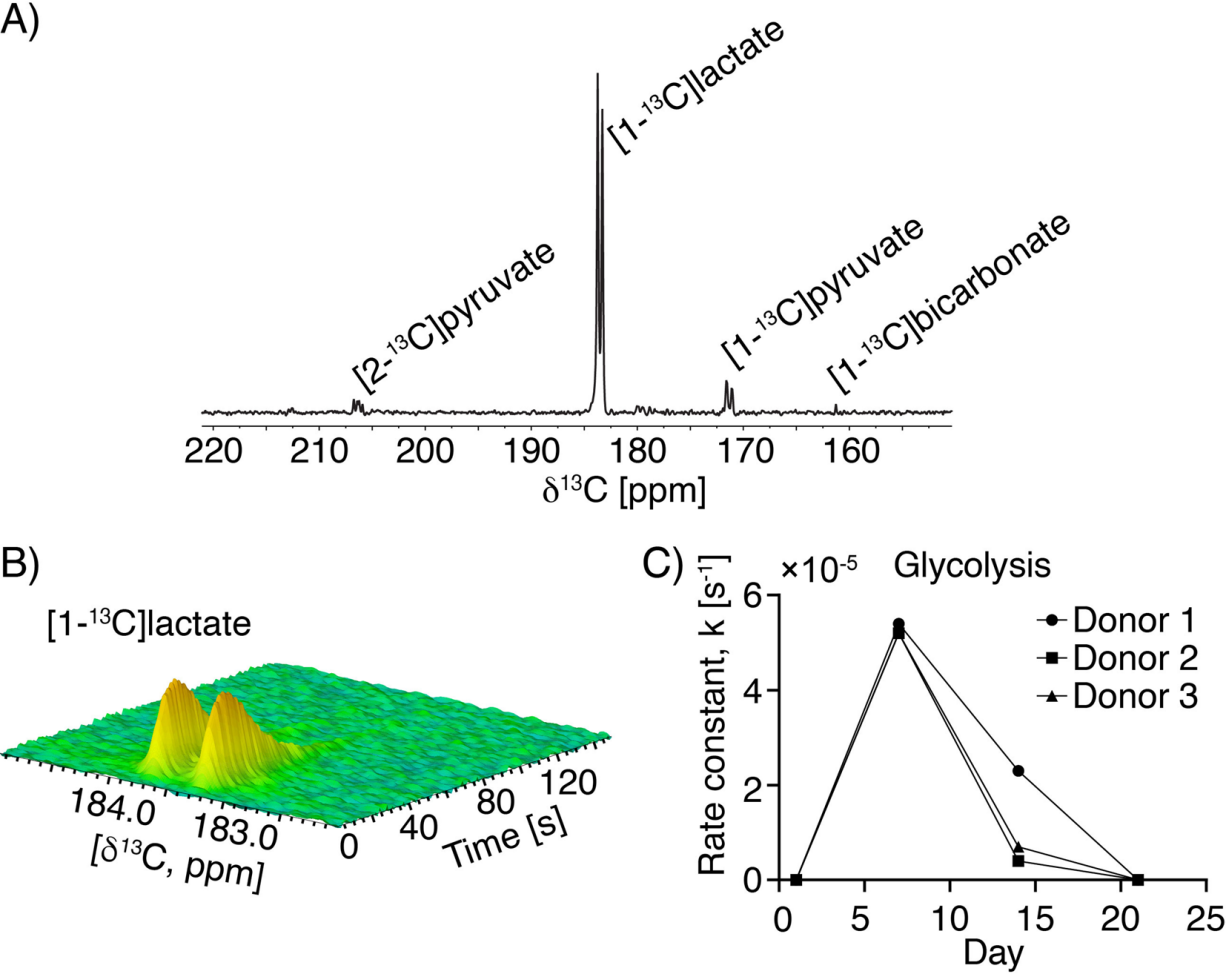
dDNP-NMR measurements of hyperpolarized [U-^13^C,^2^H]glucose conversion to lactate in CAR T cells. (**A**) Representative image of the first 50 summed spectra from day 7 activated CAR T cells. Hyperpolarized [2-^13^C]pyruvate is visible at 208 ppm, [1-^13^C]lactate at 183.5 ppm, [1-^13^C]pyruvate at 171 ppm, and [1-^13^C]bicarbonate at 162 ppm. (**B**) Representative time series showing lactate buildup from hyperpolarized [U-^13^C,^2^H]glucose conversion in CAR T cells on day 7. (**C**) Calculated rate constants for glucose-to-lactate conversion at different time points during expansion (*N* = 3 donors, 1 × 10^7^ CAR T cells per measurement). The average conversion rate on day 7 (k = (5.28 ± 0.001) × 10^− 5^ s^− 1^ was significantly higher than on day 14 (k = (1.146 ± 1.022) × 10^-5^ s^− 1^) by paired two-tailed t-test (*p* = 0.016).

The real-time glycolytic flux of CAR T cells was measured at multiple time points (day 1, 7, 14, and 21) on CAR T cells derived from three independent donors using hyperpolarized [U-^13^C,^2^H]glucose. In a single spectrum, no other metabolites besides [1-^13^C]lactate were observed (as shown in Fig. S2). However, when the first 50 spectra were summed, additional metabolites became visible: hyperpolarized [2-^13^C]pyruvate at 208 ppm, [1-^13^C]lactate at 183.5 ppm, [1-^13^C]pyruvate hydrate at 172 ppm, and [1-^13^C]bicarbonate at 162 ppm, as illustrated in Fig. 4A.

CAR T cells exhibited high metabolic conversion of hyperpolarized [U-^13^C,^2^H]glucose to [1-^13^C]lactate on day 7, similar to what was observed for activated T cells at day 5 (Fig. 4B). On day 1, glycolytic flux was undetectable, falling below the detection capabilities of hyperpolarized NMR spectroscopy. It increased to the highest production of [1-^13^C]lactate at day 7 whereafter it declined on day 14 and day 21 (Fig. 4C). The apparent glycolytic rate constant was determined on day 7 and 14 which revealed a significant 4.6-fold decrease (5.278 ± 0.001) × 10^-5^ s^− 1^ and (1.146 ± 1.022) × 10^-5^ s^− 1^ on day 7 and day 14 respectively, (*p* = 0.016, fitted curves are shown in Fig. S3 together with R^2^ values). The apparent T1 was 9 ± 1 s and 17 ± 3 s for [U-^13^C,^2^H]glucose and [1-^13^ C]lactate, respectively (nominal flip angle 20°, TR = 1 s). The increased standard deviation at day 14 reflects biological variability between donors as metabolic phenotypes diverge during the later expansion phase, whereas glycolytic activity at day 7 was consistently high and reproducible across donors. The glycolytic activity was low on day 1 and 21, which rendered the rate constant incalculable at these two timepoints, using the dynamic curves. By summation of the spectra in the dynamic curves, a higher signal-to-noise ratio was obtained. This was done on the first 50 spectra from the dynamic series, which allowed quantification of [1-^13^C]lactate on day 7, 14, and 21. Using this less precise method, we found that glycolytic flux was reduced eightfold from day 14 to day 21. In the summed spectra, [1-^13^C]lactate could not be quantified on day 1, indicating that glycolytic flux is even lower than on day 21. This corresponds to at least a 30-fold increase in glycolytic flux from day 1 to day 7, using the flux on day 21 as the lowest level of quantification.

To complement the glucose-based analysis, hyperpolarized [1-^13^C]pyruvate was employed in instances of diminished glycolytic activity. Glucose sits higher in the metabolic pathway and provides a comprehensive view of glycolytic flux. Whereas pyruvate benefits from a longer signal lifetime and the abolished ^1^*J* ^13^C-^13^C coupling. On day 21 CAR T cells metabolized hyperpolarized [1-^13^C]pyruvate to [1-^13^C]lactate with an apparent rate of (1.33 ± 1.20) × 10^-5^ s^− 1^. A comparison of pyruvate conversion on day 21 with that on day 8 revealed a decrease by a factor of 5.6 (Fig. S4). Notably, on day 21, pyruvate conversion remained detectable even though the glucose flux was not detectable, suggesting that low-level glycolysis persisted. The absence of glucose metabolism, therefore, is likely to reflect detection limits rather than a true cessation in activity.

### Nutritional availability is limited in the upstart of CAR T cell expansion

**Fig. 5:**
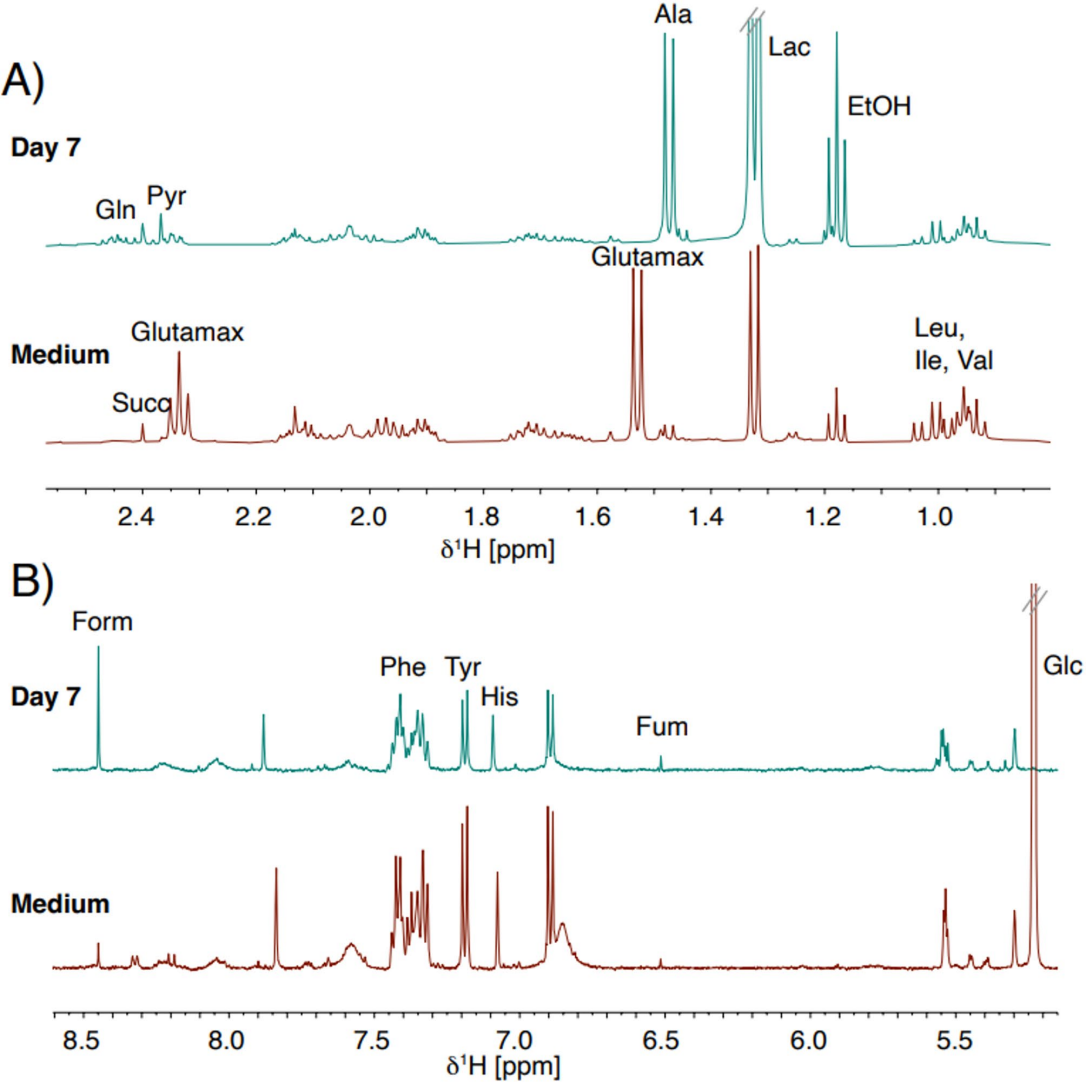
Exometabolome from ^1^H NMR. Representative 500 MHz ^1^H NMR spectra from day 7 of activated CAR T cells (blue) and fresh medium (red). (**A**) Spectral region 0.8–2.6 ppm and (**B**) Spectral region 5.2–8.6 ppm. Signals used for quantification of the following metabolites are indicated: Alanine (Ala), Ethanol (EtOH), Formate (Form), Fumarate (Fum), Glucose (Glc), GlutaMAX^™^, Glutamine (Gln), Histidine (His), Isoleucine (Ile), Lactate (Lac), Leucine (Leu), Phenylalanine (Phe), Pyruvate (Pyr), Succinate (Succ), Tyrosine (Tyr), and Valine (Val). One peak was selected for quantification of each metabolite.

To gain a comprehensive view of CAR T cell metabolism, we combined hyperpolarized NMR, which measures flux through a specific pathway, with ^1^H NMR metabolomics of spent culture media, which profiles broader nutrient changes over time. This approach links real-time metabolic activity with longer-term shifts in nutrient use, providing a more complete picture of metabolic dynamics during CAR T cell expansion. By collecting supernatant samples during routine medium changes, we used ^1^H NMR to monitor the nutritional state of the cells during the expansion protocol. Sixteen metabolites were quantified from the fresh and spent culture medium (Fig. 5).

**Fig. 6:**
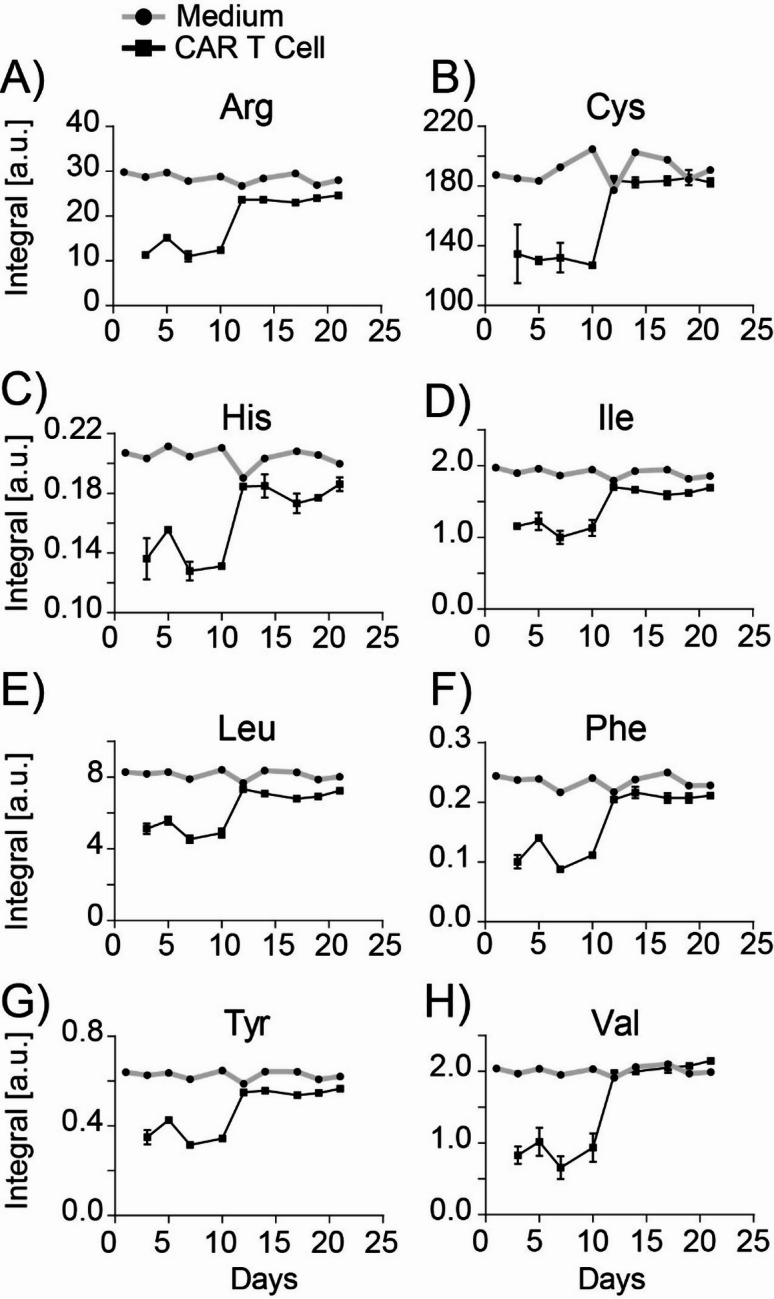
Amino Acid availability in expanded CAR T cells. Amino acid content deduced from ^1^H NMR spectra of supernatant samples from CAR T cells before feeding, compared with fresh medium samples taken on the same day. *N* = 3 biological CAR T cell replicates from the same donor expanded independently. The amino acids are shown in the following order: (**A**) Arginine (Arg), (**B**) Cysteine (Cys), (**C**) Histidine (His), (**D**) Isoleucine (Ile), (**E**) Leucine (Leu), (**F**) Phenylalanine (Phe), (**G**) Tyrosine (Tyr), (**H**) Valine (Val).

Analysis of the cell supernatants revealed significant consumption of all amino acids present in the growth medium, including arginine, cysteine, histidine, isoleucine, leucine, phenylalanine, tyrosine, and valine, during the first 12 days. After day 14, amino acid consumption was minimal (Fig. 6). This pattern reflected the high biosynthetic demands during the early stages of CAR T cell activation. The ^1^H NMR data represent absolute concentrations in the supernatant and were not normalized to cell number. Relative cell expansion over time is shown in Fig. 3F for reference. All groups of amino acids were consumed extensively. Arginine and cysteine have been reported as key contributors to polyamine synthesis and redox balance^30,31^. The branched-chain amino acids (isoleucine, leucine, and valine) were particularly consumed, reflecting their role in protein synthesis and energy production while phenylalanine and tyrosine consumption indicated active protein biosynthesis during the proliferative phase^32^.

After day 14, amino acid consumption was minimal, suggesting a metabolic shift as CAR T cells transitioned from a highly proliferative state to a more quiescent maintenance phase.

**Fig. 7:**
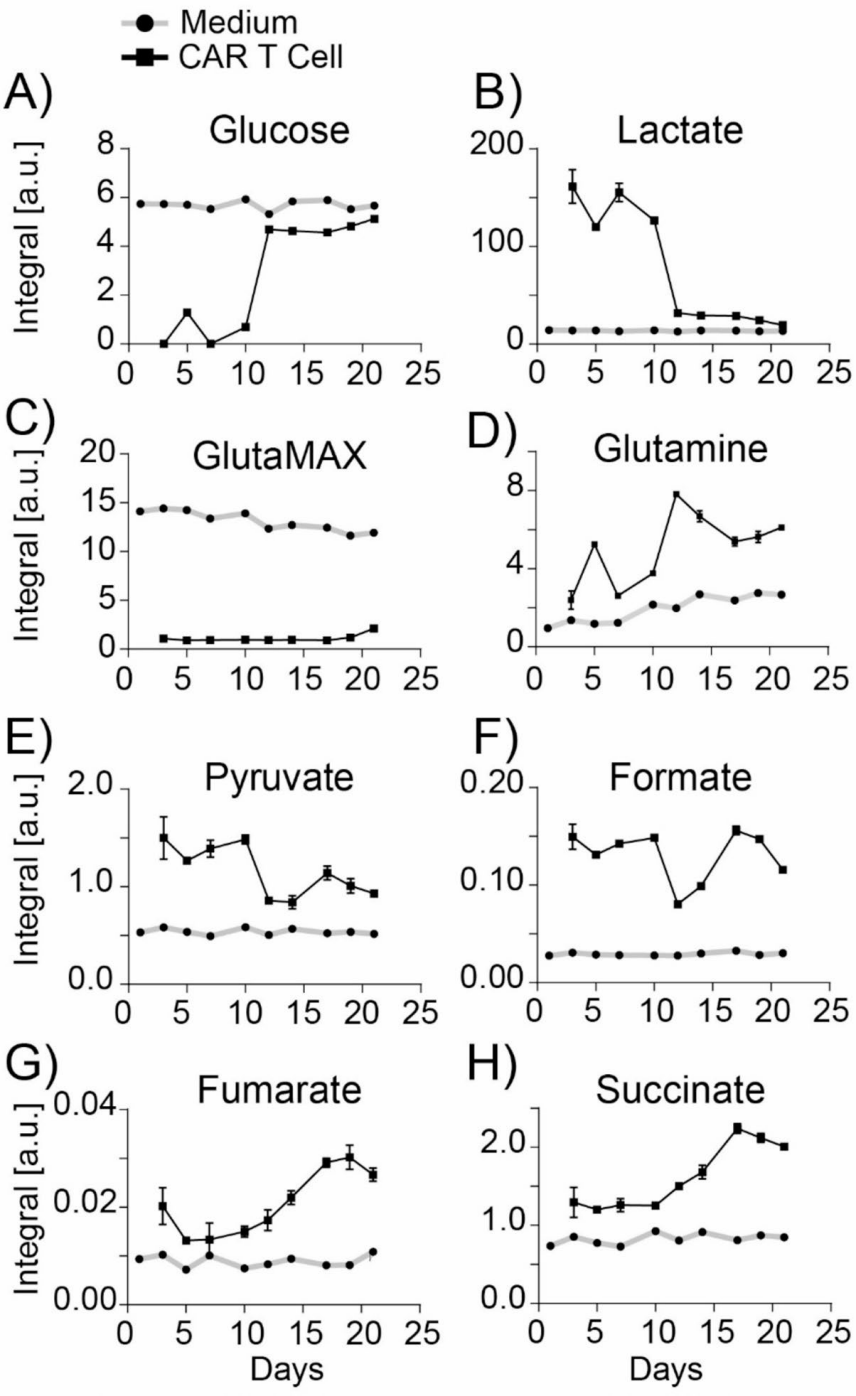
Carbon source-relevant metabolites. Carbon source-relevant metabolites deduced ^1^H NMR spectra of supernatant samples from a CAR T cell triplicate before feeding, compared with blank media samples taken on the same day. *N* = 3 biological CAR T cell replicates from the same donor expanded independently. Metabolites are shown in the following order: (**A**) Glucose, (**B**) Lactate, (**C**) GlutaMAX^™^, (**D**) Glutamine, (**E**) Pyruvate, (**F**) Formate, (**G**) Fumarate, (**H**) Succinate.

Likewise, analysis of metabolites from the central carbon metabolism revealed near-complete glucose utilization within the first 10 days (Fig. 7A), indicating glucose starvation. This observation suggested the prevalence of glucose-starved T cells when cultured under standard laboratory conditions. A particularly steep change in glucose consumption was observed between days 10 and 12 where the consumption of glucose decreased from 88% to 17% of the added amount. Similarly, stabilized glutamine provided as GlutaMAX^™^ was entirely cleaved throughout the 21-day expansion period (Fig. 7C), reflecting rapid conversion into glutamine and alanine (Fig. S5). Glutamine levels exhibited a dynamic fluctuation pattern. Low on day 3, increasing by day 5, decreasing from day 7 to 10, and rising again from day 12 onward (Fig. 7D). The high glutamine consumption between day 7 and 10 indicated a period of metabolic reprogramming in CAR T cells, characterized by elevated demands for bioenergetic and biosynthetic processes. Lactate production was substantial during the first 12 days, decreasing significantly thereafter (Fig. 7B), corroborating the glycolytic activity observed with hyperpolarized [U-^13^C,^2^H]glucose. Concurrently, pyruvate accumulated in the cell media during the first 12-day period (Fig. 7E), suggesting that CAR T cells produced excess pyruvate, which is excreted into the medium as part of their metabolic adaptation. Post day 14, an increase in TCA cycle intermediates, succinate, and fumarate, was observed (Fig. 7H and G), demonstrating a metabolic transition from aerobic glycolysis to oxidative phosphorylation. Notably, fluctuating levels of formate on day 3 to day 10 were observed, accompanied by a subsequent rise after a decline on day 12 (Fig. 7F). Formate, a key metabolite in the one-carbon metabolism, is involved in several cellular processes, ranging from nucleotide synthesis to redox balance modulation^33^. The fluctuations observed in amino acid availability, glucose utilization, lactate production, and TCA cycle intermediates collectively signify a comprehensive metabolic reprogramming occurring at distinct stages of the CAR T cell life cycle. The study used a standard CAR T cell expansion protocol where cells were diluted to 0.5 × 10^6^ CAR T cells every second day during expansion. The near-complete depletion of glucose and substantial consumption of amino acids within the first 10 days underscore the importance of ensuring sufficient nutrient availability during early culture stages to support optimal cell growth and function.

## Discussion

Metabolic profiling of CAR T cells during cell expansion is crucial, as it provides detailed insights that can aid improved function and stability of CAR T cell products used in treating hematological cancers^16,18,34,35^. In this study we first established hyperpolarized [U-^13^C,^2^H]glucose as a non-invasive molecular marker for glycolytic flux determination in CAR T cells. Previous studies have used hyperpolarized [1-^13^C]pyruvate and found a 3.6-fold increase in lactate production in activated human CD4^+^ T cells compared to their non-activated counterparts^21^. Hyperpolarized [1-^13^C]pyruvate has also been used to detect inflammatory states in M1 and M2 mice macrophages^36^. The best approach to retrieve kinetic information from hyperpolarized data is still debated. Simpler models that focus on accurate determination of the rate constant is favored, while T1 relaxation is acknowledged as a factor of signal depletion but is not considered determinant of metabolic activity. In this study, we used an approach where the contribution of the pulse angle was accounted for after fitting the rate constant. T1 values for hyperpolarized [U-^13^C,^2^H]glucose and resulting [1-^13^C]lactate were 14 and 41 s, respectively. T1 values for hyperpolarized [1-^13^C]pyruvate and resulting [1-^13^C]lactate were 38 and 17 s, respectively. Previous studies of T cells with hyperpolarized [1-^13^C]pyruvate did not report T1 values^21,36^. The T1 values for the substrates [U-^13^C,^2^H]glucose and [1-^13^C]pyruvate are in accordance with literature values^37–40^, while the T1 values for [1-^13^C]lactate differed significantly depending on which substrate it was formed from. A range of T1 values have been reported for [1-^13^C]lactate in the literature at 9.4–11.7 T (13–40 s)^37–40^. While the hyperpolarized substrate is well-defined in the extracellular space, the intracellular environment can affect the T1 of metabolic products, as higher viscosity tends to reduce T1^41^. Care must be taken when interpreting T1 values of metabolic products, since they can be influenced by metabolic pathways, transport processes, and cellular homeostasis. Here, we interpret the well-defined rate-constant, k.

Hyperpolarized [1-^13^C]pyruvate is the golden standard for non-invasive flux measurements with hyperpolarization in cells and humans. Pyruvate is a key metabolite at the end of glycolysis and is often used as a proxy for glycolytic activity. Here we for the first time demonstrated that hyperpolarized [U-^13^C,^2^H]glucose is applicable as a metabolic probe in CAR T cells and T cells. Glucose is the first metabolite in the glycolytic pathway, and it feeds into multiple processes like the pentose phosphate pathway, and oxidative phosphorylation^42^. Using hyperpolarized [U-^13^C,^2^H]glucose is therefore desired as it reflects the full glycolysis and not only the last enzymatic step in the glycolysis between pyruvate and lactate. Lactate formation was measured in both untransduced and CAR transduced T cells using hyperpolarized [U-^13^C,^2^H]glucose. In CAR T cells, we did not observe any intermediates in the glycolytic pathway upstream of pyruvate. This could indicate that CAR T cells have a different cell homeostasis compared to prostate cancer cells (PC-3), breast cancer cells (MCF-7), *E. coli* (Gram negative) bacteria, *L. Lactis* (Gram positive) bacteria and *S. cerevisiae* (yeast), all of which showed dihydroxyacetone phosphate (DHAP), 3-phosphoglycerate (3-PG) and pyruvate as glycolytic bottlenecks upon injection of hyperpolarized [U-^13^C,^2^H]glucose^25,40,43^. For CAR T cells only a small amount of bicarbonate was observed, again differentiating from previous findings in other cell types. Bicarbonate either origins from reaction in the pentose-phosphate pathway or it is produced by the pyruvate dehydrogenase complex when pyruvate enters the TCA cycle. This indicates low activity of these two pathways combined on day 7. Further studies are needed to understand the implications of what appears to be a rather unique cell homeostasis in CAR T cells. Comparable glycolytic flux was measured in T cells and CAR T cells (Figs. 1B and 4C). Since recombinant protein expression can impose a metabolic burden if the protein is too large or highly expressed, the lack of differences suggests that CAR integration did not substantially impact the overall metabolic state of the cells, as indicated by previous reports^29,44^. Our metabolomic data showed that glucose was almost completely depleted in the full period between days 3–10 (Fig. 7A), while dDNP-NMR data indicated minimal metabolic activity on day 1, which increased more than 30-fold on day 7 (Fig. 4B). Together these results demonstrated a metabolic trajectory in CAR T cells, characterized by a transition from oxidative phosphorylation (OXPHOS) to aerobic glycolysis during activation (days 1–7). The glycolytic surge on day 7 coincided with maximal cellular proliferation and activation marker expression (Fig. 3), consistent with prior reports emphasizing the role of aerobic glycolysis providing rapid energy during T cell activation^45–47^. Glycolytic flux, nutrient depletion, and activation marker expression peaked at different times, illustrating how dDNP-NMR, metabolomics, and flow cytometry capture complementary, non-synchronous aspects of CAR T cell expansion. Flow cytometry provided essential context by confirming activation status and proliferation dynamics through expression of CD69, CD25, PD-1, and other markers.

Previous ^13^C-NMR studies have reported a 6-fold increase in glycolysis within two hours of T cell activation and a 15-fold increase in glucose utilization from 48 to 96 h in activated T cells compared to quiescent T cells^18^. Notably, the study found that 85% of the utilized glucose was converted to lactate, underscoring an early metabolic shift towards anaerobic respiration^18^. Seahorse assays further supported this trend showing a 4-fold increase in extracellular acidification rate (ECAR) of CD4^+^ T cells 24 to 72 h post-activation^16^. Moreover, glucose transport in thymocytes was found to increase by 20- to 40-fold during the transition from a quiescent to an activated state, while primed T lymphocytes exhibited a 20- to 50-fold increase in glycolysis^48,49^. We observed a more than 30-fold increase in glycolytic flux measured with hyperpolarized [U-^13^C,^2^H]glucose from days 1–7. This glycolytic activation was comparable to these previous studies and confirmed that dDNP-NMR using hyperpolarized [U-^13^C,^2^H]glucose was in line with literature findings and that it reflected the transition from OXPHOS to aerobic glycolysis.

Notably, the very low glycolytic flux detected on day 1 and day 21 likely reflects both biological and technical factors. At these points, T cells adopt a predominantly oxidative metabolism, and glycolytic activity falls below the dynamic detection threshold of our method.

One of the most striking observations in our study was the distinct metabolic shift occurring days 10–12. This transition was characterized by notable changes in glucose utilization, amino acid consumption, and TCA cycle intermediate levels. In this period glucose utilization for example decreased from 88% to 17% of the available glucose (Fig. 6A). This sharp metabolic shift was partly cooperated in the proliferation data which revealed a slow linear increase days 3–10, followed by a sharp rise day 10–12 and followed by a subsequent decline days 13–15 (Fig. 3F). Despite the similar flat proliferation rates days 3–10 and days 13–15, the metabolic profile was significantly different. Amino acid consumption dropped markedly (Fig. 6A–H), lactate production decreased (Fig. 7B), and TCA cycle intermediate levels increased (Fig. 7G and H). This metabolic shift revealed that nutrient consumption did not correlate directly with proliferation i.e. biomass formation and the observations thus highlighted a disconnect between cell proliferation and glycolytic activity. During early activation, we observed significant consumption of arginine and glutamine, both of which play crucial roles in T cell metabolism. Arginine supports activation and persistence^50^, while glutamine serves as an energy source and precursor for biosynthetic pathways^51^. As glutamine is converted into glutamate and subsequently into α-ketoglutarate (α-KG), it feeds into the TCA cycle, supporting oxidative phosphorylation and ATP production^52,53^. This metabolic reprogramming may be driven by the glycolysis-mTORC1 axis, which is known to regulate nutrient sensing and metabolic adaptation^51^. Glutamine’s ability to sustain IFN-γ production, support T-effector cell ATP concentrations, and enhance antitumor activity under low-glucose conditions further underscored its critical role in CAR T cell culture conditions^51,54,55^. The observed shifts in amino acid consumption, coupled with increase in the TCA intermediates succinate and fumarate, underscored a distinct metabolic reprogramming event around day 10–12.

A major challenge identified in this study was the prolonged glucose starvation experienced by CAR T cells during the first 10 days of culture. Based on the cell proliferation profile, this depletion of nutrients was surprising. It can potentially induce metabolic stress, which likely impairs CAR T cell expansion, functional persistence, and antitumor efficacy. These effects have previously been documented, and glucose deprivation was shown to impair T cell activation and reduce cytokine production, thereby limiting T cell effector function^47,54,56^. As glucose becomes limited, cells may rely more heavily on amino acids such as glutamine^51,54,55^. However, our data showed that glutamine was also rapidly consumed. Our findings highlight that nutrient depletion, specifically glucose, arginine, and glutamine, may hinder optimal CAR T cell functionality. The depletion of these key nutrients could exacerbate metabolic stress, potentially exhaust the CAR T cells and impair their antitumor activity.

CAR T cells were expanded with CD3/CD28 beads, which provided a standardized means to define baseline metabolic kinetics and nutrient bottlenecks, but these findings potentially also establish a foundation for investigating how different CAR constructs behave when interacting with their antigen. Especially different co-stimulatory domains shape CAR T cell metabolism, as an example CD28 signaling is associated with glycolysis and effector differentiation, whereas 4-1BB promotes oxidative phosphorylation and supports memory formation^29^. Other co-stimulatory domains may show additional, unique metabolic programing, and this technique can be applied to optimize combinations and to benchmark emerging designs against established CAR constructs in future applications.

Based on these findings, several potential interventions could improve CAR T cell culture conditions. These include implementing dynamic, tailored nutrient feeding strategies that align with the evolving metabolic demands during expansion, particularly by increasing glucose, glutamine, and key amino acids in the early proliferative phase. Additionally, bioreactor systems with controlled nutrient delivery and waste removal could mitigate nutrient depletion, reduce metabolic stress, and enhance final product quality and functional persistence.

Although this study focused on *in vitro* expansion, systemic nutrient levels in vivo are more stable due to host regulation. However, the tumor microenvironment remains metabolically hostile, characterized by nutrient depletion, hypoxia, and acidosis. CAR T cells entering this environment would likely experience altered metabolic kinetics and suppressed glycolytic activity, underscoring the importance of strategies that enhance metabolic resilience both during manufacturing and post-infusion.

While metabolomics is fast and easy to perform, the combination with dDNP-NMR was powerful and complementary. Without the glycolytic flux measurements from dDNP-NMR one could interpret the metabolomics profile from day 3–10 as pure aerobic glycolysis i.e. Warburg metabolism but the combination with hyperpolarized flux data highlighted the nuanced relationship between glycolytic flux and nutrient availability.

## Conclusion

This study established the value of hyperpolarized [U-^13^C,^2^H]glucose and metabolomics for longitudinal studies of metabolic profiling in CAR T cells, providing detailed insights into their dynamic energy demands and substrate utilization. By identifying critical metabolic bottlenecks, such as glucose starvation and amino acid consumption, we highlight actionable strategies for optimizing CAR T cell manufacturing and therapeutic efficacy. These findings pave the way for further research into metabolic interventions and underscore the potential of metabolic monitoring in advancing CAR T cell therapy.

## Methods

All materials were obtained from Sigma-Aldrich unless otherwise stated.

### Collection of healthy donor T cells

This study was carried out in accordance with the Declaration of Helsinki. Human peripheral blood was obtained anonymized from healthy adults, from the Central Blood Bank, Rigshospitalet, Copenhagen, Denmark, following informed written consent from the donors. This study is approved by the Institutional review board of Rigshospitalet National Hospital (Rigshospitalet National Hospital approval BC-40).

### CAR T cell constructs and virus production

A standard second-generation anti-CD19 CAR with 41BB co-stimulatory domain was used.

Lentiviral particles that encode the CAR construct were produced using HEK293T cells. Cells were transfected with a third-generation lentiviral system, consisting of the CAR-encoding transfer plasmid along together with three helper plasmids: pMDLg/pRRE (gag/pol), pRSV-Rev (rev), and pMD2.G (VSV-G envelope). Transfection was performed using the Thermo Fisher Lipofectamine 3000 Transfection kit (Catalog number L3000015) in accordance with manufacturer’s directions, and cells were incubated at 37 °C and 5% CO_2_. Lentiviral supernatant was collected at 24 and 48 h post-transfection, filtered through a 0.45 μm filter, and concentrated using the Takara Lenti-X Concentrator (catalog number 631478) following the manufacturer’s instructions.

### T cell expansion

T cells were isolated from PBMC’s using the EasySep^™^ Direct Human T cells Isolation Kit (Catalog # 19661) and resuspended in Gibco^™^ RPMI 1640 Medium supplemented with GlutaMAX^™^ (L-alanyl-L-glutamine dipeptide, Ala-Gln), HEPES, 10% fetal bovine serum (Catalog number: 26140079), and 1% Penicillin-Streptomycin (Catalog number: 15140122), along with 20 IU/mL of IL-2 (Catalog number # 200-02-100UG ), resulting in a concentration of 1 × 10^6^ cells/mL.

On day 0, the isolated T cells were activated using Dynabeads Human T-Activator CD3/CD28 for T Cell Expansion and Activation (Catalog number: 11161D) at a 1:1 bead to T cells ratio, following the provided standard protocol. After 24 h of activation, the T cells underwent transfection with lentivirus, created in-house, containing CD19 CAR gene, at a multiplicity of infection (MOI) of 5. Following transduction, the T cells were cultured until day 3, at which point the first media sample was collected. Subsequently, media samples were collected every 2 days for proton NMR analysis, and the cells were diluted to a concentration of 0.5 × 10^6^ cells/mL, with an additional supplementation of 20 IU/mL of IL-2. On day 6 of culture, the beads were removed from the T cell culture using a dynamag-15 magnetic rack and the culture were hereafter treated as before with dilution every 2 days to a concentration of 0.5 × 10^6^ cells/mL, with an additional supplementation of 20 IU/mL of IL-2.

### Flow cytometry

Flow Cytometry Approximately 1 × 10^6^ cells were harvested from the expansion phase and stained with antibodies to determine the expression of activation markers. To confirm the direct expression of the CD19 CAR on the cell surface, CAR T cells were stained with CD19 tetramers composed of PE-streptavidin conjugated with biotinylated CD19 protein according to Friis et al.^57^. Stained cells were washed to remove any unbound antibodies or tetramers and then fixed in 1% paraformaldehyde (PFA) overnight. Phenotypic expressions were determined by flow cytometry using an LSRFortessa instrument (BD Biosciences). Flow cytometry data were analyzed using FlowJo software. The following antibodies were used: CD69 BUV396 (catalog number 564364, clone FN50, BD Biosciences), CD25 BB700 (catalog number 566447, clone M-A251, BD Biosciences), PD-1 BV711 (catalog number 564017, clone EH12.1, BD Biosciences), CD127 BUV737 (catalog number 612795, clone HIL-M21, BD Biosciences), and CD27 BV605 (catalog number 302836, clone O323, BioLegend).

### ^1^H NMR

Media samples for ^1^H NMR were taken before dilution and centrifuged (10.000 rpm, 10 min) to remove cells in the medium. The same media bottle was used throughout the experiment and a sample of the added media was taken as a blank sample at each intervention time.

Preparation of Buffer for ^1^H NMR Analysis.

Phosphate buffer (240 mM, pH 7.5, without DSS) was prepared by dissolving NaH_2_PO_4_ in a D_2_O/Milli-Q water mixture (60%/40%) and adjusting the pH with 10 M NaOH. DSS was then added to 0.644 mM. For NMR, 500 µL of the sample was mixed with 100 µL of the prepared buffer.

1D ^1^H-NMR spectra were acquired with a NOESY pre-saturation pulse sequence (noesygppr1d) using a Bruker 500 MHz NMR instrument equipped with a 5 mm DCH CryoProbe and an 11.7 T UltraShield magnet. Spectra were acquired using a SampleExpress sample changer. The data was collected by accumulating 256 scans. Each FID was sampled by acquiring 35,416 complex data points during an acquisition time of 1.7 s and an inter scan delay (d1) of 4 s, resulting in 24 min acquisition time per sample. NMR data were processed and analyzed using MestReNova software version 14.1.1 (Mestrelab Research, E).

### dDNP-NMR experiments

Experiments were conducted following protocols described previously^58^. Shortly, substrate sample stock solutions were prepared using [1-^13^C]pyruvic acid and deuterated [U-^13^C,^2^H]glucose in water (1:0.8 w/w) respectively. The [1-^13^C]pyruvic acid solution was doped with the trityl radical AH111501 (GE Healthcare) to a final concentration of 17 mM and Gadoteridol gadolinium chelate solution (Bracco Imaging) to 1.5 mM. The glucose solution was doped with OX063 to a concentration of 22 mM and with Gadoteridol to 2 mM. Approximately 4.5 mg of the pyruvic acid preparation or 31 mg of the glucose stock preparation were hyperpolarized using the Hypersense 3.3 T polarizer (Oxford Instruments) until equilibrium polarization was achieved, typically within 1 h.

Following polarization, each sample was rapidly dissolved in 5 mL of phosphate buffer ( pH 7.4, 40 mM). For pyruvic acid, an additional 5 µL of 10 M sodium hydroxide was added to neutralize the pH. The final temperature of the solutions was maintained at approximately 310 K. After dissolution, the concentration of [1-^13^ C]pyruvate was approximately 10 mM, and the [U-^13^C,^2^H]glucose concentration 17 mM. Equal volumes of the dissolution solution and cell media were mixed, resulting in a final concentration of approximately half for each substrate in the cell suspension. Both samples achieved a liquid state polarization of approximately 25%. The dissolved hyperpolarized substrate solution was collected at the polarizer outlet, and 400 µL was drawn into a 1 mL syringe for injection via an injection line into the NMR spectrometer.

For the injection, 1 × 10^7^ CAR T cells were first centrifuged at 500*g * for 5 min. The cell pellet was then washed with PBS containing Ca^2⁺^ and Mg^2+^ and centrifuged again at 500*g * for 5 min. Following the second centrifugation, the cells were resuspended in 150 µL of MM-DMEM (Gibco, #A1443001) supplemented with 4 mM L-Glutamine and 25 mM HEPES (Gibco, #15630-056), bringing the total volume to approximately 200 µL. The suspension was then transferred to a Shigemi NMR tube using a pipet with an elongated tip.

dDNP-NMR spectra were acquired on a Bruker 500 MHz AVANCE NEO spectrometer equipped with a 5 mm DCH cryoprobe. Transfer time between polarizer and NMR spectrometer was 9 s and acquisition was started simultaneously with the injection. A series of ^13^C NMR spectra were recorded using a nominal 20° pulse angle and 1 or 2-second delay for [U-^13^C,^2^H]glucose. For [1-^13^C]pyruvate a nominal 10° pulse angle and a 1 or 2-second delay between pulses was used. The dDNP-NMR data were processed using MestReNova software, with phase and baseline corrections performed prior to integration of the [U-^13^C,^2^H]glucose, [1-^13^C]pyruvate, and [1-^13^C]lactate signals. Integrals were obtained by integrating over one peak at different chemical shifts for each metabolite.

Reaction kinetics were analyzed by fitting a Python-based model to the integrated spectral data, using ordinary differential equations to describe the conversion dynamics of [1-^13^C]pyruvate to [1-^13^C]lactate and the apparent conversion rate from [U-^13^C,^2^H]glucose^25,58^.

1. dS/dt = -k*S(t) – 1/T1_s_*S(t) – p*S(t).
2. dP/dt = k*S(t) – 1/T1_p_*P(t) – p*P(t).

Where *p* = 1-cos(pw)/TR, pw is the pulse angle and TR is the time between pulses.

To minimize the number of fitted parameters the pulse angle (pw) was set to zero in the model. The fitted T1 values are thus apparent values which include pulsing. When handling data sets with low SNR we find that this method is more robust compared to including freedom from the flip angle due to interdependence of T1 and the pulse angle. For [U-^13^C,^2^H]glucose data on day 7 and 14, which were acquired with a TR = 1 s and nominal flip angle of 20°, the apparent T1 values were fitted to 9 ± 1 s and 17 ± 3 s for [U-^13^C,^2^H]glucose and [1-^13^C]lactate respectively. After correction for a pulse angle of 15° this corresponds to a T1 of 14 s and 41 s for [U-^13^C,^2^H]glucose and [1-^13^C]lactate, respectively. For the [1-^13^C]pyruvate data on day 14 and 21, which were acquired with a TR = 1 and nominal flip angle of 10°, the apparent T1 values were fitted to 28 ± 2 s and 15 ± 1 s for [1-^13^C]pyruvate and [1-^13^C]lactate, respectively. After correction for a pulse angle of 8° this corresponds to a T1 of 38 s and 17 s for [1-^13^C]pyruvate and [1-^13^C]lactate, respectively.

### Statistics

The experiments presented in Fig. 1 serve as representative examples demonstrating the method’s capabilities and typical signal evolution of pyruvate and glucose (*N* = 1). In contrast, Figs. 3 and 4 show data obtained from three biological replicates derived from healthy donor CAR T cells, expanded independently under identical conditions. Metabolomic data, including amino acid and glucose depletion, were collected from three replicates of the same donors to ensure consistency and minimize inter-donor variability (Figs. 6 and 7). For continuous variables, such as metabolic rate constants (k) derived from dDNP-NMR, amino acid concentrations, and glucose depletion rates, data were expressed as mean ± standard deviation (SD).

Rate constants for glycolysis and pyruvate-to-lactate conversion were calculated by fitting experimental data to differential equations using a least-squares optimization model implemented in Python. Goodness-of-fit was evaluated using the coefficient of determination (R^2^) and residual analysis.

For ^1^H NMR metabolomics, integrals of metabolite peaks were normalized against internal standards (DSS) and fresh medium controls to account for experimental variability.

Statistical significance of rate constant changes between day 7 and day 14 (*p* < 0.05) was assessed using paired two-tailed t-tests in GraphPad Prism 10.

## Supplementary Information

Below is the link to the electronic supplementary material.

Supplementary Material 1

## Data Availability

Data for this article, including raw data from dDNP-NMR, 1 H NMR and flow cytometry are available at Zenodo at 10.5281/zenodo.15044319.

## Abbreviations

CAR: Chimeric antigen receptor
CAR T: Chimeric antigen receptor T cell
CD: Cluster of differentiation
dDNP-NMR: Dissolution dynamic nuclear polarization nuclear magnetic resonance
ECAR: Extracellular acidification rate
FBS: Fetal bovine serum
GLUT1: Glucose transporter 1
IL-2: Interleukin-2
LDH: Lactate dehydrogenase
MOI: Multiplicity of infection
mTOR: Mammalian target of rapamycin
NAD+/NADH: Nicotinamide adenine dinucleotide (oxidized/reduced)
NMR: Nuclear magnetic resonance
OCR: Oxygen consumption rate
OXPHOS: Oxidative phosphorylation
PBS: Phosphate buffered saline
PBMCs: Peripheral blood mononuclear cells
PI3K: Phosphoinositide 3-kinase
RPMI: Roswell Park Memorial Institute (cell culture medium)
TCA: Tricarboxylic acid cycle
TCR: T cell receptor
TME: Tumor microenvironment
