# Supplementary material for: Mapping real-time metabolic kinetics of expanded CAR T cells using hyperpolarized 13C-glucose and metabolomics
Source: Sci Rep. 2025 Nov 20;15:40929. doi: 10.1038/s41598-025-24712-2 (PMC12635404; doi:10.1038/s41598-025-24712-2)
Supplement: Supplementary file 1 — Supplementary Material 1 [file 41598_2025_24712_MOESM1_ESM.pdf]

Supplementary Information

Mapping real-time metabolic kinetics of expanded CAR T cells using Hyperpolarized ^{13}C-glucose and metabolomics

Thomas B.W. Mathiassen[1#], Mikkel Rasmus Hansen[2#], Magnus Karlsson[1], Sine Reker Hadrup[1], Maria Ormhøj[2*], Pernille Rose Jensen[1*]

[1]Section for Magnetic Resonance, Department of Health Technology, Technical University of Denmark, 2800 Kgs. Lyngby, Denmark.

[2]Section for Experimental and Translational Immunology (xTI), Department of Health Technology, Technical University of Denmark, 2800 Kgs. Lyngby, Denmark.

[#]Contributed equally to this publication.

*Corresponding authors

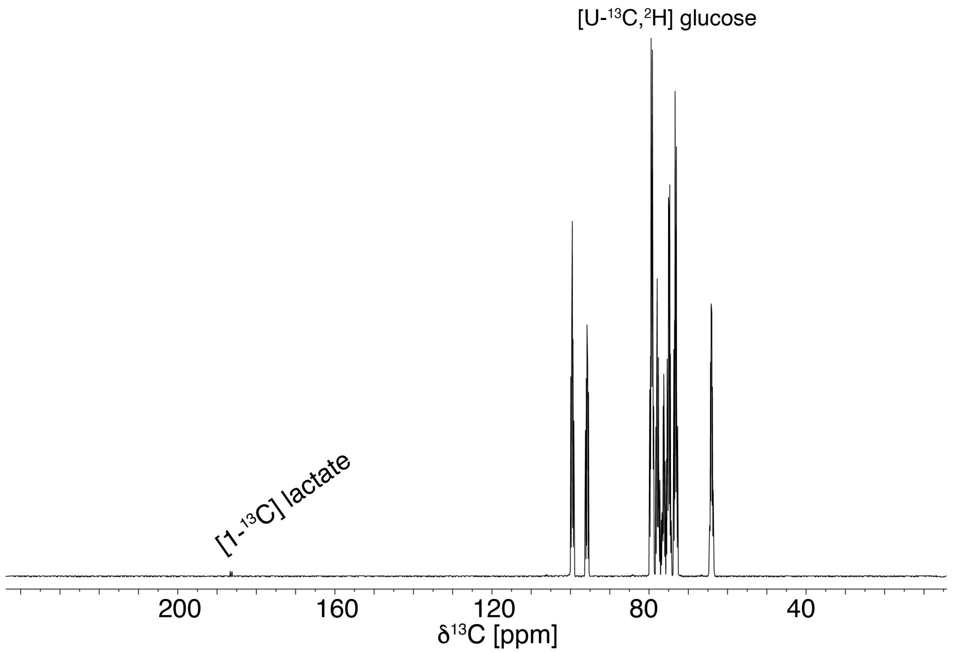

Figure S1: Single full spectrum of hyperpolarized [U-^{13}C,^{2}H]glucose from a dynamic series taken at day 7 where the [1-^{13}C]lactate was highest.

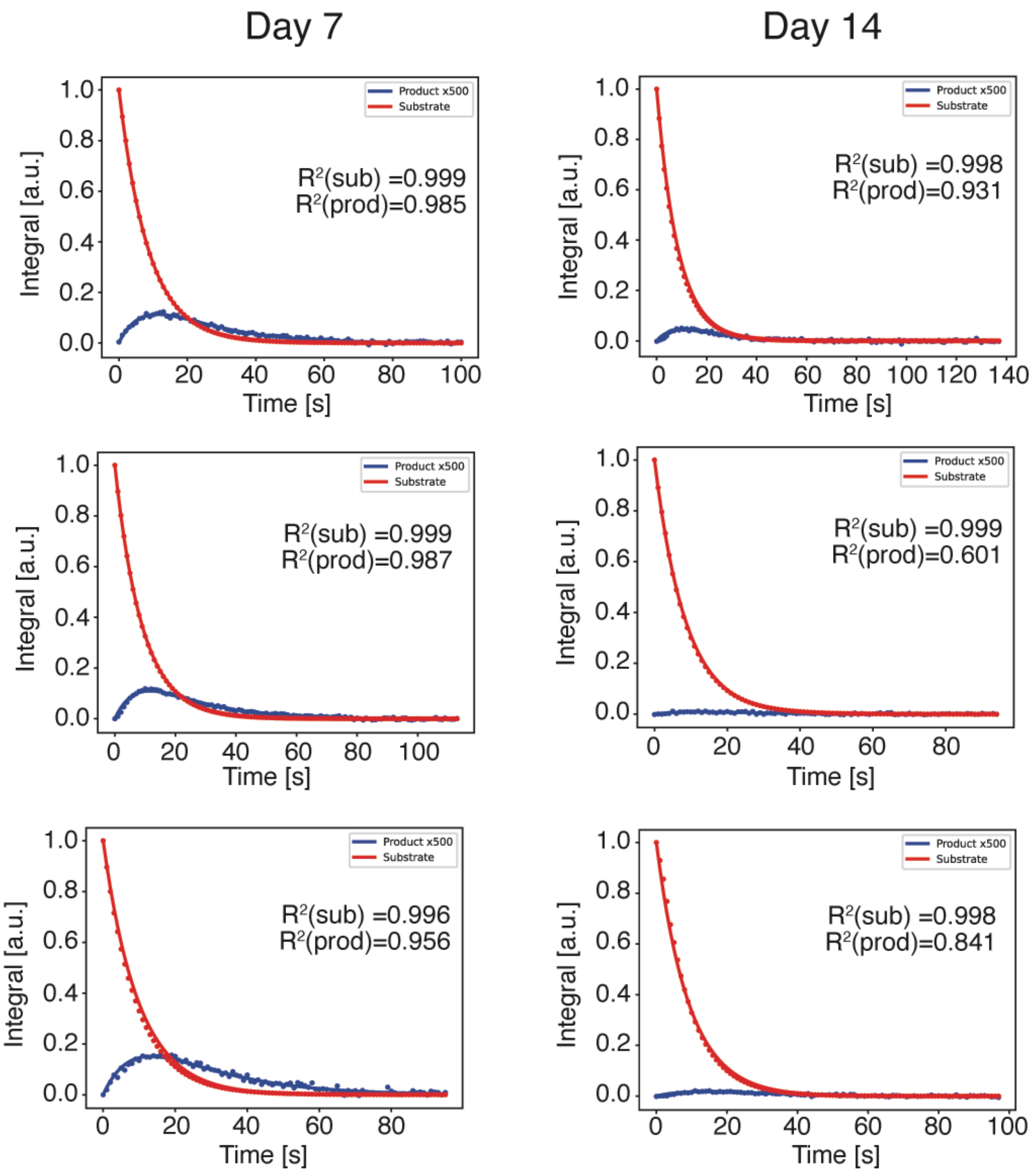

Figure S2: CAR T cell metabolism measured using hyperpolarized [U-13C,2H]glucose on day 7 and 14. Conversion of [U-^{13}C,^{2}H]glucose to [1-^{13}C]lactate in 1 × 10^7 CAR T cells at different time points post-activation. Fitted rate constants are presented in Figure 4C.

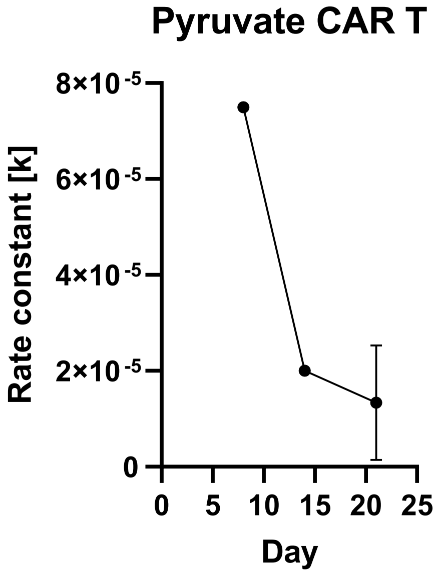

Figure S3: CAR T cell metabolism measured using hyperpolarized pyruvate on day 7, 14, and 21. Conversion of [1-^{13}C]pyruvate to [1-^{13}C]lactate in 1×10^7 CAR T cells at different time points post-activation. Fitted rate constants are day 7 $k = 7.5 \times 10^{-5}$ s^{-1}, day 14 $k = 2.0 \times 10^{-5}$ s^{-1}, and day 21 $k = 1.33 \pm 1.20 \times 10^{-5}$ s^{-1}.

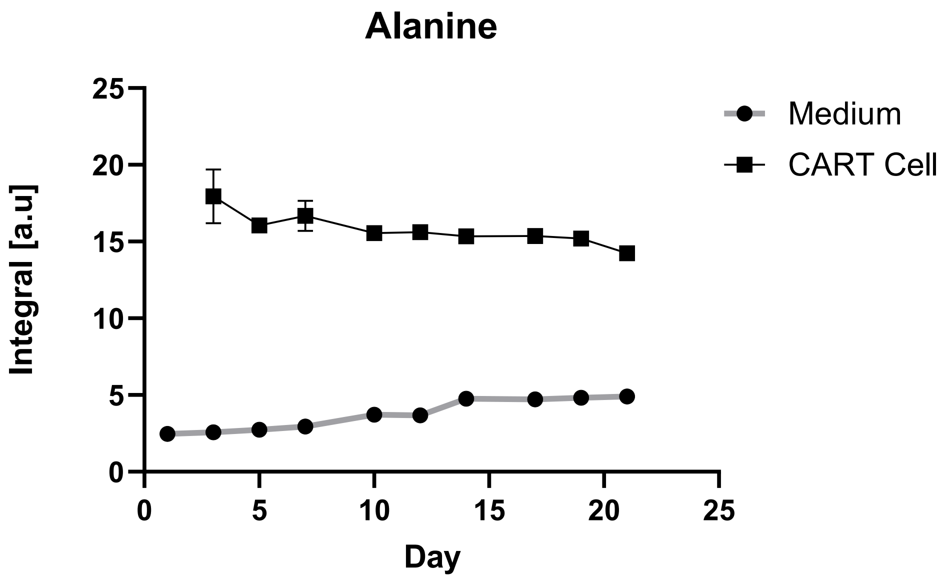

Figure S4: Alanine availability in expanded CAR-T cells. Amino acid content deduced from ^{1}H NMR spectra of supernatant samples from CAR T cells before feeding, compared with fresh medium samples taken on the same day. N=3 biological CAR-T cell replicates from the same donor expanded independently. Alanine is present as part of GlutaMAX™, a dipeptide of alanine and glutamine. The gradual increase of alanine in medium-only samples results from the slow spontaneous cleavage of GlutaMAX™ into its components. In contrast, GlutaMAX™ is fully cleaved in CAR-T cell samples, but alanine consumption remains minimal compared to the substantial glutamine uptake.

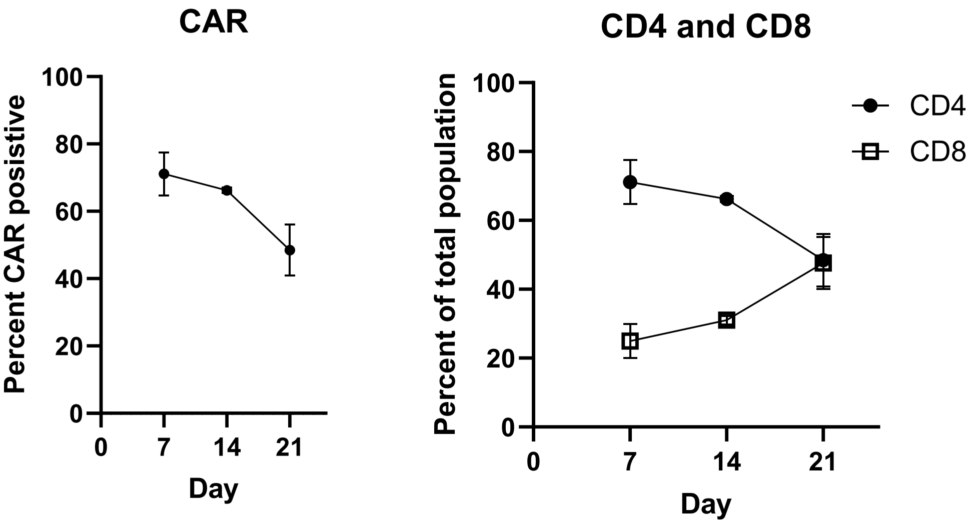

Figure S5. CAR expression and CD4/CD8 composition in CAR T cells over time.

Primary human T cells from the three independent donors. CAR expression was determined by GFP positivity encoded downstream of a T2A element in the vector insert. CD4 and CD8 subsets were identified by flow cytometry using BV480-conjugated anti-CD4 and PerCP-conjugated anti-CD8 antibodies. (Left) The percentage of CAR-expressing T cells decreased gradually from day 7 to day 21 post-transduction. (Right) The composition of the CAR T cell population shifted over time, with $CD4^{+}$ cells dominating early after transduction, followed by a relative increase in CD8 cells by day 21. Data represent mean ±SD of the three donors.
